# Evaluation of Thermal and Mechanical Properties of Bi-In-Sn/WO_3_ Composites for Efficient Heat Dissipation

**DOI:** 10.3390/ma17215315

**Published:** 2024-10-31

**Authors:** Die Wu, Zhen Ning, Yanlin Zhu, Rui Yuan

**Affiliations:** 1 Institute for Mathematical and Computational Materials Science, Chengdu Advanced Metal Materials Industry Technology Research Institute Co., Ltd., Chengdu 610300, China; zhenning410@163.com (Z.N.); ali.yanlinzhu@outlook.com (Y.Z.); 2 State Key Laboratory of Vanadium and Titanium Resources Comprehensive Utilization, Chengdu 610031, China; 3 Engineering State Key Laboratory of Polymer Materials Engineering, College of Polymer Science, Sichuan University, Chengdu 610500, China; 13088017929@163.com

**Keywords:** Bi-In-Sn/WO_3_ composite, phase change materials, thermal interface materials, mechanochemistry

## Abstract

Phase change materials (PCMs) offer promising solutions for efficient thermal management in electronic devices, energy storage systems, and renewable energy applications due to their capacity to store and release significant thermal energy during phase transitions. This study investigates the thermal and physical properties of Bi-In-Sn/WO_3_ composites, specifically for their use as phase change thermal interface materials (PCM-TIMs). The Bi-In-Sn/WO_3_ composite was synthesized through mechanochemical grinding, which enabled the uniform dispersion of WO_3_ particles within the Bi-In-Sn alloy matrix. The addition of WO_3_ particles markedly improved the composite’s thermal conductivity and transformed its physical form into a putty-like consistency, addressing leakage issues typically associated with pure Bi-In-Sn alloys. Microstructural analyses demonstrated the existence of a continuous interface between the liquid metal and WO_3_ phases, with no gaps, ensuring structural stability. Thermal performance tests demonstrated that the Bi-In-Sn/WO_3_ composite achieved improved thermal conductivity, and reduced volumetric latent heat, and there was a slight increase in thermal contact resistance with higher WO_3_ content. These findings highlight the potential of Bi-In-Sn/WO_3_ composites for utilization as advanced PCM-TIMs, offering enhanced heat dissipation, stability, and physical integrity for high-performance electronic and energy systems.

## 1. Introduction

In recent years, phase change materials (PCMs) have attracted significant attention for their remarkable potential to store and release substantial amounts of thermal energy during phase transitions [1]. This unique property makes PCMs particularly effective for thermal management across various fields, including energy storage, electronics, and renewable energy systems [2,3,4,5,6,7]. With the increasing power and compactness of electronic devices, efficient thermal regulation has become vital to prevent overheating and ensure optimal performance. PCMs offer a promising solution to address these emerging challenges [8]. Traditional thermal interface materials (TIMs) often fail to meet the increasing requirements for enhanced thermal conductivity and mechanical strength [9]. To overcome these limitations, PCMs have been shown to be a cutting-edge alternative, employing their latent heat properties to provide improved heat management and thermal performance [10,11].

Among the diverse range of PCMs, Bi-In-Sn alloys have been recognized for their favorable properties, including low melting points, high thermal conductivity, and strong mechanical stability [12]. The favorable properties of Bi-In-Sn alloys make them highly desirable for applications in electronics, power devices, and advanced energy systems [13,14]. Their ability to undergo solid-to-liquid phase transitions enables them to absorb significant amounts of heat, making them particularly effective in managing sudden thermal spikes [15]. However, a critical limitation of pure Bi-In-Sn alloys lies in their tendency to flow uncontrollably upon melting, which can lead to leakage and significantly compromise efficiency [16]. This presents a significant challenge in terms of the practical use of Bi-In-Sn alloys in real-time thermal management. To overcome these limitations, materials such as tungsten trioxide (WO_3_) films have been investigated for energy storage and electrochemical applications, where they offer benefits like corrosion resistance and improved performance in supercapacitors and fuel cells [17]. Incorporating materials like WO_3_ can enhance the stability and performance of systems where Bi-In-Sn alloys alone may not be sufficient.

To overcome this limitation, integrating WO_3_ particles into the Bi-In-Sn alloy matrix presents a promising strategy. The incorporation not only increases the thermal conductivity of the composite but also mitigates leakage by altering the physical state of the material during melting, thus enhancing overall stability and performance. Specifically, as the Bi-In-Sn alloy melts, the incorporation of WO_3_ particles transforms the molten material into a paste-like consistency. This change effectively eliminates the risk of fluid leakage, ensuring greater stability and reliability during thermal management [18,19]. The unique aspect of this approach is the mechanochemical integration of WO_3_ particles, which allows for intimate atomic-level interactions between the metal alloy and the WO_3_ phase [20]. Unlike conventional methods, mechanochemistry creates a continuous interface between Bi-In-Sn and WO_3_, improving thermal conductivity and mechanical stability while preserving the material’s structural integrity. The modification enhances the composite’s mechanical properties, enabling it to perform reliably even in high-temperature conditions, thereby transforming it into a durable solution for challenging applications [21].

Mechanochemistry is essential for achieving a successful integration of WO_3_ particles within the Bi-In-Sn alloy, facilitating optimal interaction and performance between the components [22,23]. The approach optimizes the interaction between the alloy and the particles, leading to a uniform dispersion of WO_3_ within the alloy matrix [24]. Such consistent distribution is essential for achieving enhanced thermal and mechanical performance. This method also offers a scalable and environmentally friendly approach to composite development by minimizing the need for solvents and extreme processing conditions commonly employed for material synthesis. Furthermore, the incorporation of WO_3_ particles improves the stability of the alloy during thermal cycling, allowing it to endure repeated phase changes while maintaining performance integrity without significant degradation [25].

The current study investigates the thermal and mechanical properties of Bi-In-Sn/WO_3_ composites, emphasizing their potential application as phase change thermal interface materials (PCM-TIMs) [26,27]. It also explores the effects of incorporating WO_3_ particles on the thermal conductivity, phase change behavior, and long-term stability of the alloy. Furthermore, the study evaluates the composite’s effectiveness in overcoming typical challenges faced by phase change materials, including leakage, thermal resistance, and maintaining structural integrity throughout phase transitions [28,29]. The results of the current study highlight the potential of Bi-In-Sn/WO_3_ composites as advanced thermal interface materials, particularly for high-performance electronics, power devices, and other energy-intensive applications that require efficient and reliable heat dissipation [30].

This study aims to investigate the integration of WO_3_ particles into Bi-In-Sn alloys to improve their thermal properties for use in phase change thermal interface materials (PCM-TIMs). Specifically, it evaluates how WO_3_ incorporation affects the composite’s thermal conductivity, phase change behavior, and long-term thermal stability. This research also addresses common challenges in phase change materials, such as leakage and thermal resistance during phase transitions, to explore a more efficient and reliable solution for high-performance electronics, power devices, and energy systems.

## 2. Experimental Section

Materials: The Bi-In-Sn alloy (51 wt% indium, 32.5 wt% bismuth, 16.5 wt% tin) was purchased from Shengte Xincai Electronic Technology Co., Ltd., Changsha, China and Spherical WO_3_ particles were purchased from Beijing High-tech New Material Technology Co., Ltd., Beijing, China.

Preparation of Bi-In-Sn/WO_3_ composite: Spherical WO_3_ powder with a particle size of 5 μm was used to prepare the Bi-In-Sn/WO_3_ composite. The Bi-In-Sn alloy, which has a melting point of 71 °C, was placed into an agate mortar and then heated in an oven set at 100 °C, a temperature above the alloy’s melting point. The alloy was held at this temperature for 20 min to ensure that it was fully molten. Once fully liquefied, the pre-measured WO_3_ powder was added to the molten alloy. Various mixtures were prepared with different mass ratios of WO_3_ to Bi-In-Sn (0.1:0.9, 0.15:0.85, 0.2:0.8, 0.25:0.75, 0.3:0.7, 0.35:0.65, 0.4:0.6, and 0.45:0.55). The mixture was manually ground for 30 min in the agate mortar to achieve the uniform dispersion of WO_3_ within the alloy matrix. During grinding, the temperature was held at 100 °C to keep the Bi-In-Sn alloy in its molten state. The WO_3_-to-Bi-In-Sn ratios were selected based on findings from similar composite systems [24]. When the WO_3_ content is below 0.1, the composite remains fluid after remelting, making it more susceptible to leakage. Conversely, with WO_3_ content above 0.5, the composite takes on a powder-like form, hindering structural cohesion. Ratios between 0.1 and 0.5 were thus chosen to achieve a putty-like consistency, optimizing thermal performance and structural integrity. Following grinding, the mixture was cooled to solidify the Bi-In-Sn/WO_3_ composite, enhancing its thermal properties and phase transition behavior.

Characterization: The morphological features of the Bi-In-Sn/WO_3_ composite were assessed using a scanning electron microscope (SEM, FEI Quanta 650, Hillsboro, OR, USA) in conjunction with energy dispersive spectroscopy (EDS). The Bi-In-Sn/WO_3_ composite was cut with a double-focused ion beam (FIB, FEI Helios G4 CX, Hillsboro, OR, USA), and the elemental distribution in its cross-section was characterized using a high-resolution transmission electron microscope (HRTEM, FEI Talos F200X TEM, Hillsboro, OR, USA) equipped with an energy dispersive spectrometer (EDS). The structural properties of the material were investigated using X-ray diffraction (XRD) and X-ray photoelectron spectroscopy (XPS). Thermal diffusivity measurements were conducted with a laser flash analyzer (LFA467, NETZSCH, Selb, Germany). The thermal resistance (Ri) of the composite under varying pressures was evaluated using the Longwin 9389 steady-state heat-flow method, following ASTM D5470 standards [31]. The volume-specific enthalpy of the Bi-In-Sn/WO_3_ composite was determined via differential scanning calorimetry (DSC, NETZSCH DSC 214 Polyma, Selb, Germany), a technique that evaluates heat flow during phase transitions to derive enthalpy values.

## 3. Results and Discussion

### 3.1. Synthesis of Bi-In-Sn/WO_3_ via Mechanochemistry

**Figure 1:**
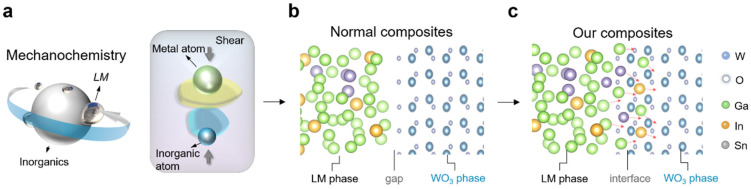
Schematic illustration of synthesis and microstructure of Bi-In-Sn/WO_3_ composite via mechanochemistry. (**a**) Mechanochemical process between LM (Bi-In-Sn) and WO_3_ particles; (**b**) Normal composites showing gaps at the interface between LM and WO_3_ phases; (**c**) Our Bi-In-Sn/WO_3_ composite showing a continuous, gap-free interface. Note: Color-coded elements include W (blue), O (light blue), Ga (green), In (yellow), and Sn (orange). Arrows indicate the direction of atomic interaction under shear.

The Bi-In-Sn/WO_3_ composite was successfully synthesized using mechanochemistry, employing a high-energy grinding technique to facilitate interactions between the Bi-In-Sn liquid metal (LM) alloy and the WO_3_ phase. The specific details of the materials and preparation process, including the size of the WO_3_ particles (5 μm) and the melting point of the Bi-In-Sn alloy (71 °C), are provided in the “Preparation of Bi-In-Sn/WO_3_ composite” section. As illustrated in Figure 1a, the mechanochemical process enabled metal atoms from the Bi-In-Sn alloy to endure shear-induced diffusion, allowing them to penetrate the WO_3_ lattice. The method promoted intimate contact between the LM phase and WO_3_, facilitating atomic-level interactions.

In the traditional composite shown in Figure 1b, a noticeable gap was observed between the liquid metal (LM) and WO_3_ phases, reducing the thermal and mechanical performance. However, the mechanochemical synthesis method applied in this study achieved a continuous interface between the LM and WO_3_ phases, as shown in Figure 1c. The Bi-In-Sn atoms effectively diffused into the WO_3_ lattice structure, eliminating the gaps commonly observed in the conventional composite. The diffusion of Bi, In, and Sn into the WO_3_ crystal structure resulted in a stable, tightly bonded interface.

The interaction between the liquid metal and WO_3_ strengthened the chemical bonds at the microscopic level, greatly enhancing the overall stability of the Bi-In-Sn/WO_3_ composite [24]. Furthermore, the absence of gaps between the LM phase and WO_3_ phase is anticipated to enhance thermal conductivity and mechanical properties, thereby enhancing the overall performance of the Bi-In-Sn/WO_3_ composite as thermal interface material. The direct interaction between the LM phase and the WO_3_ phase ensures a uniform distribution of stress and heat, resulting in enhanced phase change characteristics with improved thermal management capabilities.

### 3.2. Microstructure and Physical State of Bi-In-Sn/WO_3_ Composite

**Figure 2:**
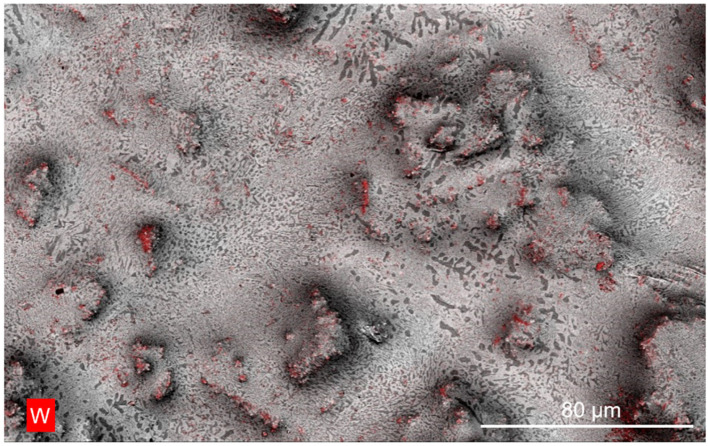
SEM image of Bi-In-Sn/WO_3_ composite. The red-highlighted areas represent tungsten (W) distribution in the composite.

The microstructure of the Bi-In-Sn/WO_3_ composite was characterized using scanning electron microscopy (SEM) as presented in Figure 2, demonstrating the uniform dispersion of WO_3_ particles throughout the Bi-In-Sn matrix. The WO_3_ particles presented a spherical shape and were uniformly dispersed throughout the Bi-In-Sn matrix, with no significant agglomeration detected. The uniform distribution demonstrated the effectiveness of the mechanochemical synthesis method, which plays a vital role in enhancing the thermal and mechanical properties of the material. This uniform dispersion is essential for enhancing the composite’s thermal cycling performance, as it enables consistent heat distribution and prevents localized thermal stress during repeated phase transitions. As a result, the material’s structural integrity is preserved throughout prolonged thermal cycling, leading to improved stability and durability in practical applications.

**Figure 3:**
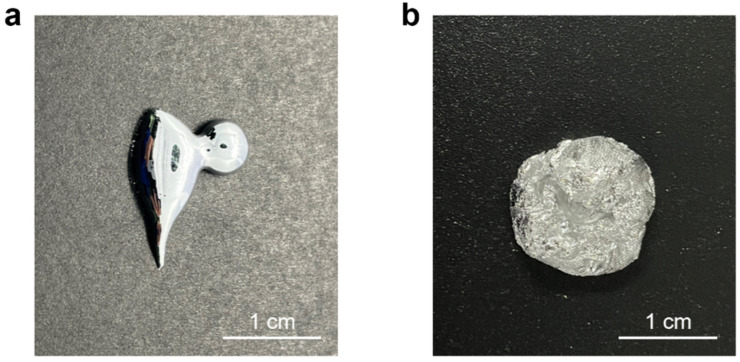
Optical images of (**a**) pure Bi-In-Sn; and (**b**) Bi-In-Sn/WO_3_ composite.

The incorporation of WO_3_ significantly altered the physical state of the Bi-In-Sn alloy. Figure 3 shows this transformation by comparing the pure Bi-In-Sn alloy (Figure 3a) with the Bi-In-Sn/WO_3_ composite (Figure 3b). The pure Bi-In-Sn alloy showed high fluidity, posing potential leakage challenges in practical applications. However, the incorporation of WO_3_ altered the physical state of the alloy, transforming it from a liquid to a more putty-like, semi-solid form. The change significantly reduced the risk of leakage, enhancing the composite’s suitability for use as an effective thermal interface material.

The putty-like nature of the Bi-In-Sn/WO_3_ composite enhanced its structural stability during phase transitions, while still facilitating efficient heat transfer. The uniform dispersion of WO_3_ throughout the composite reinforces the Bi-In-Sn alloy, stabilizing its structure and mitigating the uncontrolled flow seen in the pure alloy. The improvement in physical stability is a key factor in optimizing the composite’s functionality as a thermal interface material, especially in demanding applications where maintaining structural integrity under thermal cycling and mechanical stress is essential for long-term performance.

### 3.3. Phase Analysis and Bonding in Bi-In-Sn/WO_3_ Composite

**Figure 4:**
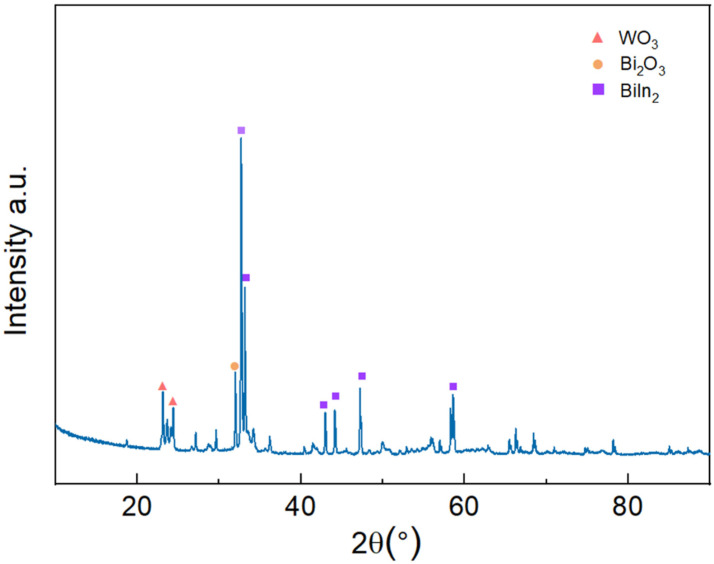
X-ray diffractogram of Bi-In-Sn/WO_3_ composite.

The phase composition of the Bi-In-Sn/WO_3_ composite was analyzed using X-ray diffraction (XRD). As illustrated in Figure 4, the XRD pattern revealed the distinct presence of both Bi-In-Sn and WO_3_ phases, confirming that no new alloy phases were formed during the synthesis process. The absence of intermetallic alloy phases is essential for the long-term stability of the composite in thermal interface applications. In contrast to alloys like Ga-In-Sn, which tend to develop rigid intermetallic phases upon contact with metals including Cu, Ag, or Al [32], the Bi-In-Sn/WO_3_ composite preserves its putty-like consistency over time, as shown in Figure 3b. This characteristic is critical for preventing the increased thermal resistance often associated with hardening in other materials.

**Figure 5:**
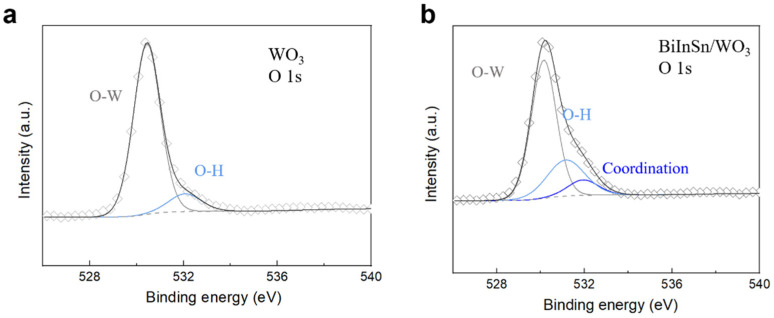
XPS analysis of (**a**) WO_3_, and (**b**) Bi-In-Sn/WO_3_ composite.

The XPS analysis further confirms the stable interaction between Bi-In-Sn and WO_3_. Figure 5 compares the O 1s spectra of pure WO_3_ (Figure 5a) with that of the Bi-In-Sn/WO_3_ composite (Figure 5b). The appearance of a shifted O 1s peak revealed the formation of a coordination bond between the Bi-In-Sn alloy and WO_3_ in the composite [33,34,35]. This binding energy shift indicated the development of a stable coordination covalent bond between the alloy’s metal atoms and WO_3_’s oxygen atoms, significantly improving the structural stability of the composite. The interaction effectively prevents phase separation and degradation, ensuring the long-term performance of the composite in thermal management applications.

The formation of these coordination bonds enhanced the interaction between the liquid metal and WO_3_ phases, enabling the composite to retain its mechanical flexibility while preventing the hardening commonly observed during alloy formation. With its structure and the absence of unwanted alloy phases, the Bi-In-Sn/WO_3_ composite emerges as a highly suitable material for prolonged use in thermal interface applications. It ensures sustained low thermal resistance and preserves mechanical flexibility over extended periods of application.

### 3.4. Interface Analysis of Bi-In-Sn/WO_3_ Composite

**Figure 6:**
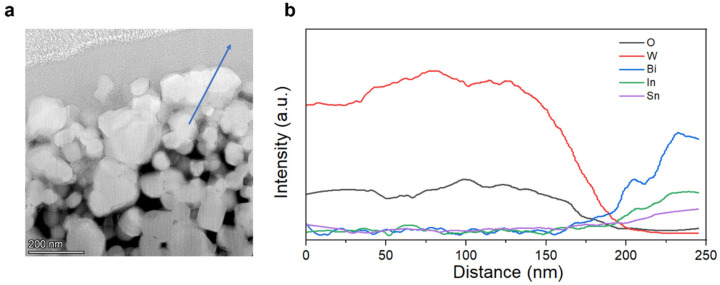
(**a**) A schematic representation of the Bi-In-Sn/WO_3_ composite following FIB cutting and HADDF imaging, with the arrow indicating the direction of the line scan; (**b**) EDS line scan showing the distribution of O, W, Bi, In, and Sn along the interface.

High-angle annular dark-field (HAADF) imaging and energy-dispersive X-ray spectroscopy (EDS) line scanning were used to characterize the interface between Bi-In-Sn and WO_3_ within the composite. Figure 6a shows the cross-sectional view of the composite obtained through focused ion beam (FIB) cutting, revealing the microstructure of the interface region. Figure 6b shows the EDS line scan across the interface, demonstrating a gradual reduction in W and O signals together with a corresponding increase in Bi, In, and Sn signals. The gradual shift in elemental concentrations suggests that Bi-In-Sn atoms have successfully diffused into the WO_3_ lattice, resulting in a smooth transition interface without abrupt changes or gaps.

The gradual change in elemental composition suggested atomic-level diffusion, confirming the formation of a coherent interface between the two phases [36]. Such interfacial bonding prevents delamination and ensures the structural integrity required for long-term stability in thermal applications. Furthermore, the continuous nature of the transition zone is essential for reducing thermal resistance and maintaining the composite’s mechanical flexibility.

**Figure 7:**
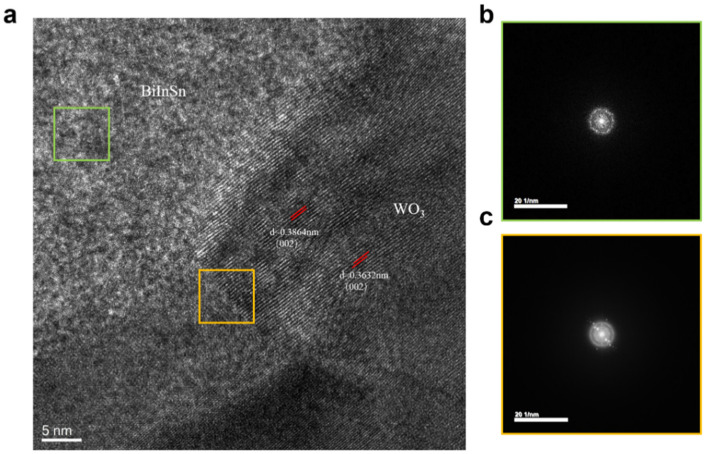
(**a**) High-resolution transmission electron microscopy image of Bi-In-Sn/WO_3_ interface, showing lattice structure. The green box highlights the Bi-In-Sn region, while the yellow box marks the transition interface with WO_3_; (**b**) SAED pattern of Bi-In-Sn (green box); and (**c**) transition interface between Bi-In-Sn and WO_3_ (yellow box).

Further evidence for the formation of the interface was obtained through high-resolution transmission electron microscopy (HRTEM) and a selected area electron diffraction (SAED) analysis. Figure 7a presents the HRTEM image of the Bi-In-Sn/WO_3_ interface, clearly illustrating the distinct boundary between the two phases. Figure 7b,c show the SAED patterns corresponding to the Bi-In-Sn region and the transition interface, respectively. The diffraction pattern at the interface revealed a combination of crystalline order and partial distortion, likely arising from the incorporation of Bi-In-Sn atoms into the WO_3_ lattice.

The observed broadening of the diffraction spots at the interface signified the lattice distortion, providing further evidence for atomic-level diffusion. The structural modification strengthens the interfacial bonding between Bi-In-Sn and WO_3_, thereby establishing a stable interface with the potential to endure mechanical stress without the formation of gaps. Ensuring a gap-free interface is essential for reducing thermal resistance, which significantly enhances the efficiency of the composite as a thermal interface material.

### 3.5. Thermal Properties of Bi-In-Sn/WO_3_ Composite

The assessment of the thermal properties of the Bi-In-Sn/WO_3_ composite involved analyzing thermal conductivity, thermal contact resistance, and volumetric latent heat relative to the mass fraction of WO_3_. These metrics are pivotal for understanding the composite’s capability as a TIM.

**Figure 8:**
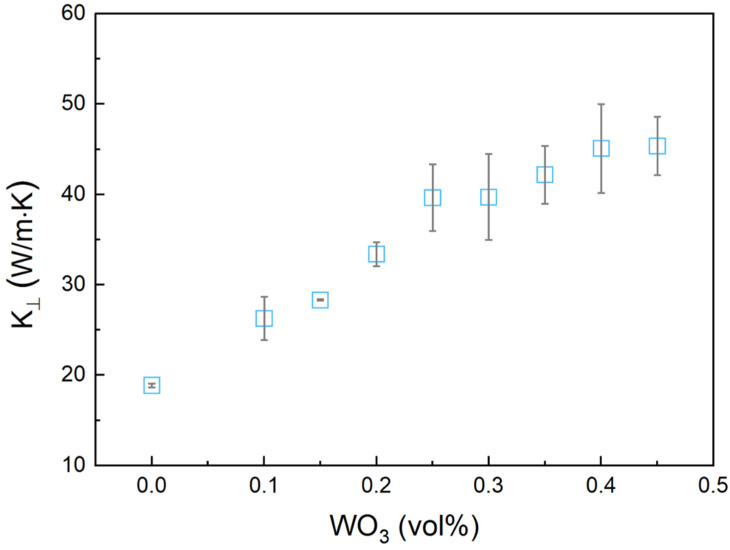
Thermal conductivity of Bi-In-Sn/WO_3_ composite as function of WO_3_ mass fraction.

Figure 8 shows the variation in thermal conductivity with the WO_3_ mass fraction. An increase in WO_3_ content led to a remarkable enhancement in thermal conductivity, likely due to the formation of continuous heat conduction pathways within the matrix. The enhancement can be attributed to the high intrinsic thermal conductivity of WO_3_, which enables more efficient heat transfer within the material. At higher WO_3_ concentrations, the material may approach a percolation threshold where interconnected WO_3_ networks are formed, which further enhances the heat transfer efficiency. Therefore, the integration of WO_3_ particles significantly enhanced the heat dissipation capability of the composite, positioning it as an optimal choice for applications that require effective thermal management. When the WO_3_ content exceeds 0.5 vol%, the composite shifts into a discontinuous, powder-like state [24], which complicates accurate thermal conductivity measurements and limits its effectiveness as a phase change thermal interface material.

**Figure 9:**
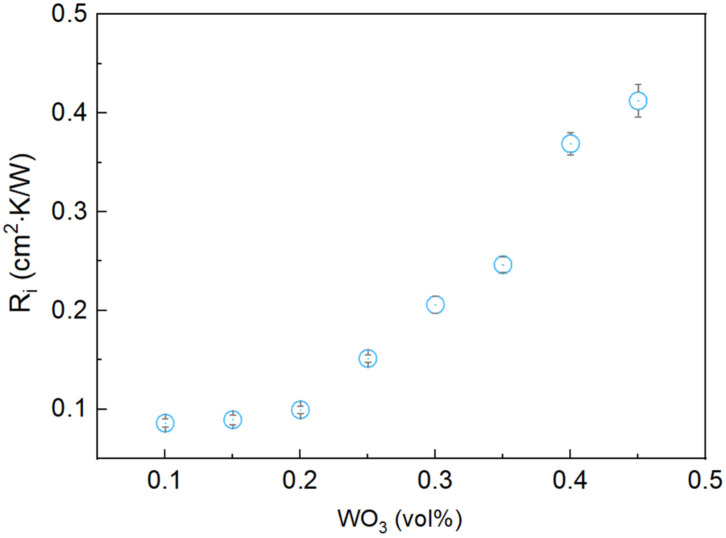
Thermal contact resistance of Bi-In-Sn/WO_3_ composite as function of WO_3_ mass fraction.

Figure 9 illustrates the variation in thermal contact resistance (Rᵢ) with an increasing WO_3_ mass fraction, demonstrating increased thermal contact resistance with increasing WO_3_ content. The observed trend can be attributed to the reduced flexibility of the composite at higher WO_3_ concentrations, which hinders its ability to conform to surface irregularities at the interface. The change in flexibility is due to the composite’s transition from a putty-like to a powder-like form as WO_3_ content increases, as described in the “Preparation of Bi-In-Sn/WO_3_ Composite” section. Although an increase in Rᵢ was observed, the overall thermal performance remained strong, largely due to the significant improvement in thermal conductivity, as demonstrated in Figure 8.

**Figure 10:**
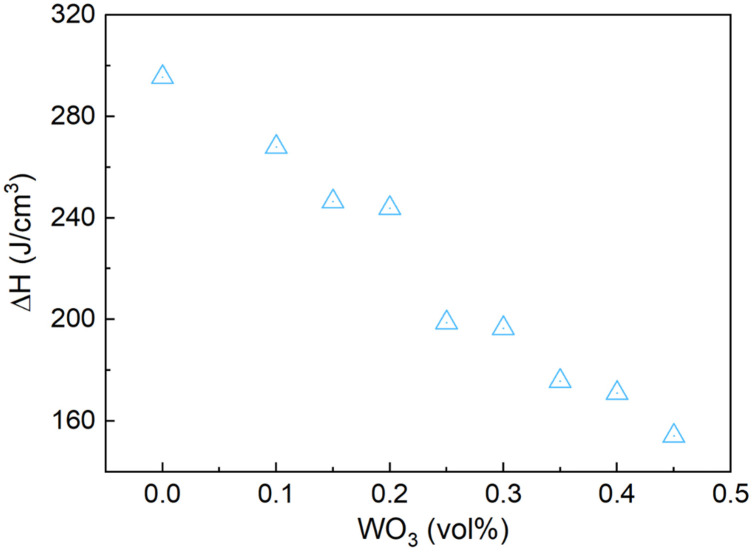
Volumetric latent heat of Bi-In-Sn/WO_3_ composite as function of WO_3_ mass fraction.

Figure 10 illustrates the relationship between the volumetric latent heat and WO_3_ mass fraction, with a decrease in volumetric latent heat observed as WO_3_ content increased. The reduction occurred because WO_3_, a non-phase change material, decreased the latent heat capacity of the Bi-In-Sn alloy. Despite the reduction in volumetric latent heat, the composite retained a sufficient capacity for heat absorption and release, making it suitable for applications that rely on phase transition processes.

**Figure 11:**
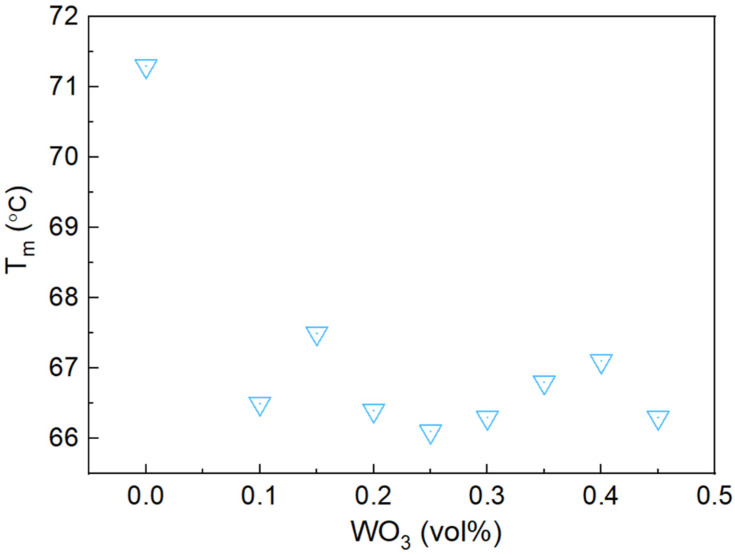
Phase transition temperature (T_m_) of Bi-In-Sn/WO_3_ composite as function of WO_3_ mass fraction.

Figure 11 shows the variation in the phase transition temperature (T_m_) of the Bi-In-Sn/WO_3_ composite relative to WO_3_ content. As illustrated, the phase transition temperature slightly decreases as WO_3_ content increases, especially once WO_3_ concentration exceeds 0.1 vol%. This reduction is likely due to the dilution effect of WO_3_—a non-phase change material—within the Bi-In-Sn matrix. The presence of WO_3_ disrupts the alloy’s uniform structure, lowering the overall phase transition temperature. Despite this decrease, the composite maintains a phase transition temperature suitable for effective thermal management applications. At higher WO_3_ concentrations, the transition temperature stabilizes, indicating that further increases in WO_3_ content have a minimal impact on the composite’s phase change properties.

## 4. Conclusions

This study successfully developed a Bi-In-Sn/WO_3_ composite as a highly efficient phase change thermal interface material (PCM-TIM). Integrating WO_3_ particles within the Bi-In-Sn alloy matrix significantly enhanced the composite’s thermal and mechanical properties. The composite achieved a thermal conductivity of up to 45 W/m·K with a WO_3_ content of 0.45, demonstrating superior heat dissipation capabilities over conventional materials. The observed thermal conductivity surpasses that of many existing PCM-TIMs, establishing it as a strong candidate for demanding thermal management applications. Thermal contact resistance (Rᵢ) was kept low, around 0.4 cm^2^·K/W, ensuring effective thermal transfer during phase transitions—a performance feature not typically observed in comparable materials. Volumetric latent heat was reduced to 160 J/cm^3^, preserving sufficient heat storage capacity despite the addition of non-phase change WO_3_ particles.

Mechanochemical synthesis facilitated the uniform dispersion of WO_3_ and fostered strong bonding between the phases, leading to enhanced thermal conductivity and mechanical stability. The composite maintained its structural integrity without forming intermetallic phases, which is essential for preserving its putty-like consistency and ensuring low thermal resistance during phase transitions. The phase transition temperature (T_m_) of the composite showed a slight decrease, from 71 °C to 66 °C, indicating a minimal yet significant thermal response as WO_3_ content increased.

The Bi-In-Sn/WO_3_ composite offers distinct advantages over traditional materials, particularly in high-performance environments where both high thermal conductivity and low thermal resistance are critical. The composite demonstrated significant potential as a PCM-TIM, providing enhanced thermal conductivity, higher structural stability, and effective heat dissipation management for high-performance electronic devices and energy systems. This composite is specifically suited for applications in power electronics, advanced cooling systems for energy devices, and high-performance computing units, where efficient heat dissipation and thermal regulation are vital for ensuring device reliability and longevity. These findings offer valuable insights for the development of next-generation thermal interface materials that can address the stringent thermal management demands of modern industries, particularly in electronics, power devices, and other energy-intensive applications.

## Data Availability

The original contributions presented in the study are included in the article, further inquiries can be directed to the corresponding author.
